# Dual-Polarized Isolation-Improved MIMO Inverted-F Antenna Through an L-Shaped Decoupler

**DOI:** 10.3390/s26102999

**Published:** 2026-05-10

**Authors:** Mohammed A. Hassan, Ahmad H. Abdelgwad

**Affiliations:** 1 Electrical Engineering Department, College of Engineering, King Faisal University, Al-Ahsa 31982, Saudi Arabia; 2 Electrical Engineering Department, Fayoum University, Fayoum 63514, Egypt; aha05@fayoum.edu.eg

**Keywords:** antenna isolation, dual-polarized antenna, MIMO, diversity, parasitic element, WLAN

## Abstract

**Highlights:**

**What are the main findings?**

- A compact two-element MIMO IFA with orthogonal orientation achieves simultaneous pattern and polarization diversity in the 2.4 GHz WLAN band, while maintaining a simple planar structure.
- An L-shaped parasitic element effectively suppresses mutual coupling, enabling excellent isolation (S_21_ < −30 dB near 2.4 GHz) and a wide impedance bandwidth of approximately 2.2–2.7 GHz, validated by measurements.

**What are the implications of the main findings?**

- The proposed design offers a low-profile, easy-to-fabricate solution for modern WLAN devices requiring high isolation and diversity performance without complex decoupling techniques.
- The demonstrated decoupling approach using a parasitic element can be extended to other compact MIMO antenna systems to enhance isolation and system capacity.

**Abstract:**

This paper introduces a compact MIMO antenna system designed for WLAN applications, offering dual polarization, strong isolation, and pattern diversity. The system includes two orthogonally positioned inverted-F antenna (IFA) elements operating in the 2.4 GHz WLAN band. To achieve polarization diversity, each element is designed and excited with a perpendicular feed. An L-shaped metallic parasitic element is placed close to the antennas to significantly reduce mutual coupling and enhance isolation. The antenna’s layout is straightforward and planar, making it easy to fabricate without requiring complex manufacturing steps. A prototype of the design was built and tested, and the experimental results show good agreement with simulated data. The fabricated antenna achieves a wide operating bandwidth from around 2.2 to 2.7 GHz and exhibits excellent port isolation, with S_21_ better than −30 dB at 2.4 GHz. The proposed antenna with L-parasitic provides an efficiency of around −0.53 dB (89%) and a peak gain of 3.3 dBi at 2.4 GHz. Further, it offers an exceptionally low envelope correlation coefficient (ECC), approximately 0.0004, and diversity gain of nearly 10 dB, ensuring robust diversity and MIMO performance. These characteristics make the proposed design a promising option for use in low-profile modern WLAN MIMO systems.

## 1. Introduction

In recent years, the rapid progression of wireless communication technologies has driven the demand for higher data rates within the constraints of limited spectral resources and power budgets. Multiple-input multiple-output (MIMO) antenna systems have gained significant attention as an effective solution to enhance data throughput and channel capacity without requiring additional bandwidth or transmission power [1,2,3]. To support practical implementation, MIMO antennas must exhibit a compact form factor, high radiation efficiency, and strong isolation between ports within the operating frequency bands.

A variety of antenna structures have been employed in MIMO designs, including inverted-F antennas (IFAs) [4], monopoles [5], and planar patch antennas [6]. Recently, an increased emphasis has been placed on MIMO systems that provide pattern and polarization diversity to improve link reliability and mitigate multipath fading effects. Such diversity techniques enhance coverage and energy efficiency by enabling adaptive signal delivery toward intended users [7].

Numerous strategies have been proposed to realize pattern and polarization diversity in compact MIMO configurations [8,9,10,11,12,13]. For example, the work in [8] utilizes a dual-fed square patch antenna with a rat-race coupler to achieve pattern reconfigurability. The design in [9] introduces two triangular notched elements to provide pattern diversity across the 3.1–5.0 GHz range. A dual-band printed dipole array operating at 1.8 GHz and 2.4 GHz is proposed in [10], using equal-amplitude, phase-shifted feeding to produce distinct radiation patterns. Both pattern and polarization diversity are achieved using parasitic elements and radiating elements orientation in [11]. In [12,13], structural diversity is achieved by combining dissimilar antenna elements. Polarization diversity is demonstrated in [12] through the use of a PIFA and a sleeve antenna, although the resulting structure lacks full planarity. A compact implementation in [13] achieves both diversity types by combining a patch element and a modified interdigital structure fed by a coplanar slot line; however, the placement of elements on opposite sides of the substrate leads to limited isolation.

Mutual coupling remains a critical design concern in MIMO systems, as it negatively impacts correlation, gain, and system capacity. Various techniques have been employed to reduce coupling [14,15], including ground plane alterations [16], parasitic elements [17], antenna reconfigurations [18], impedance matching networks [19], neutralization lines [20,21], electromagnetic band gap (EBG) structures [22,23], and decoupling circuits [24].

The primary contribution of this work is the design and experimental validation of a compact, planar two-element MIMO antenna that simultaneously provides pattern and polarization diversity for 2.4 GHz WLAN applications. Rather than employing complex decoupling networks, this work integrates a straightforward L-shaped parasitic structure that successfully mitigates mutual coupling, achieving inter-port isolation greater than 30 dB. Beyond decoupling, this parasitic element introduces an additional resonance that broadens the impedance bandwidth by 80%, covering 2.2 to 2.7 GHz. By delivering exceptional MIMO performance metrics—specifically, an envelope correlation coefficient (ECC) of approximately 0.0004 and a diversity gain approaching 10 dB—within a highly manufacturable footprint, the proposed system offers a robust and practical solution for space-constrained modern wireless networks. The rest of the paper is organized as follows. Section 2 presents the antenna configuration, parametric analysis, and simulation results, including S-parameters, radiation characteristics, efficiency, and current distribution, in addition to MIMO and diversity performance. Section 3 discusses the fabricated mockup and measurement results, and a state-of-the-art comparison is presented in Section 4. Finally, the conclusions of this study are summarized in Section 5.

## 2. MIMO Antenna Structure and Operation

### 2.1. Antenna Configuration

**Figure 1:**
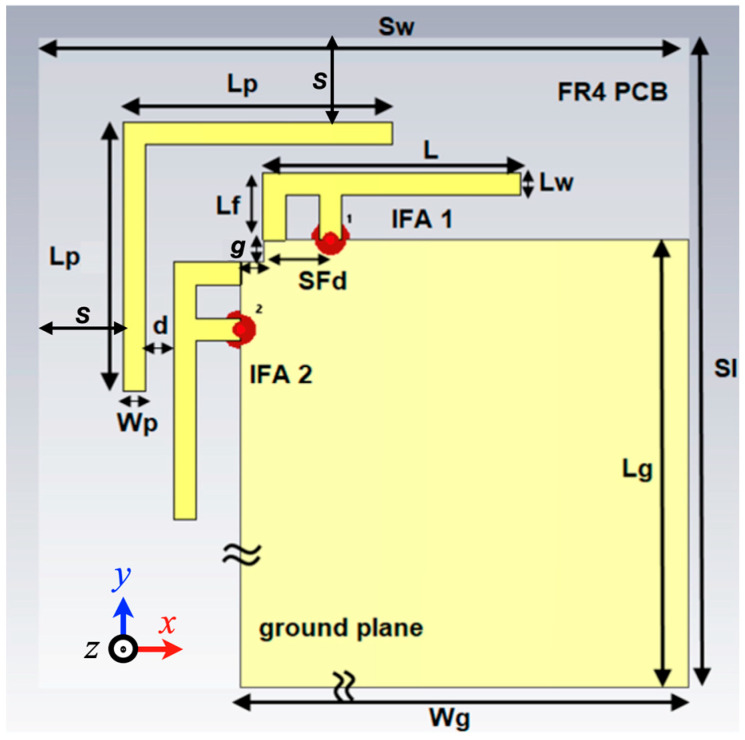
The proposed cross-polarized MIMO antenna with L-parasitic geometry. Dimensions (in mm): Wg = 100, Lg = 100, Lf = 6, Lw = 2, SFd = 5, L = 23, g = 3, S = 7.5, Wp = 2, d = 2.5, Lp = 24, Sl = 118, and Sw = 118.

Figure 1 illustrates the layout of the proposed two-element IFA-based MIMO system, which incorporates an L-shaped parasitic element to effectively reduce mutual coupling. The antenna is implemented on an FR4 substrate with a relative dielectric constant ($\varepsilon_{r}$) of 4.3 and a thickness of 0.8 mm. The overall dimensions of the structure are 118 × 118 × 0.8 mm^3^. To facilitate both pattern and polarization diversity, the two IFA elements are positioned orthogonally at the corner of the ground plane. The IFA dimensions were optimized to operate at around 2.4 GHz, where the IFA effective length ($L_{eff}$) is approximately a quarter wavelength at the resonance frequency ($f_{0}$) and is calculated using [25]:

(1) $$L_{eff}\approx \frac{C}{4f_{0}\sqrt{\varepsilon_{eff}}}\approx \frac{C}{4f_{0}\sqrt{{(\varepsilon }_{r}+1)/2}}$$

In MIMO or diversity antenna configurations, ensuring sufficient isolation between the radiating elements is a key design objective. To address this, an L-shaped metallic parasitic strip is strategically placed between the antenna elements, which not only improves isolation but also contributes to bandwidth enhancement. The presence of the parasitic structure enables the excitation of an additional resonance, thereby supporting wideband impedance matching.

### 2.2. Parametric Analysis

**Figure 2:**
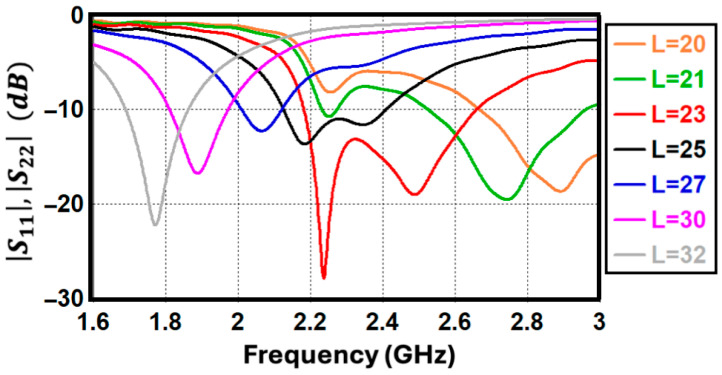
Reflection coefficient (S_11_, S_22_ in dB) of the proposed antenna for different IFA arm lengths (*L*).

To achieve optimal performance, a parametric study was conducted to evaluate the influence of critical design variables on the proposed antenna. Each parameter was analyzed independently while maintaining the remaining dimensions at their nominal values, as specified in the caption of Figure 1. Figure 2 illustrates the simulated reflection coefficient across various inverted-F antenna (IFA) arm lengths (*L*). Consistent with the theoretical relationship defined in (1), increasing the arm length shifts the resonant frequency downward. Consequently, adjusting the *L* provides an effective mechanism for tuning the antenna to the desired operating bands.

**Figure 3:**
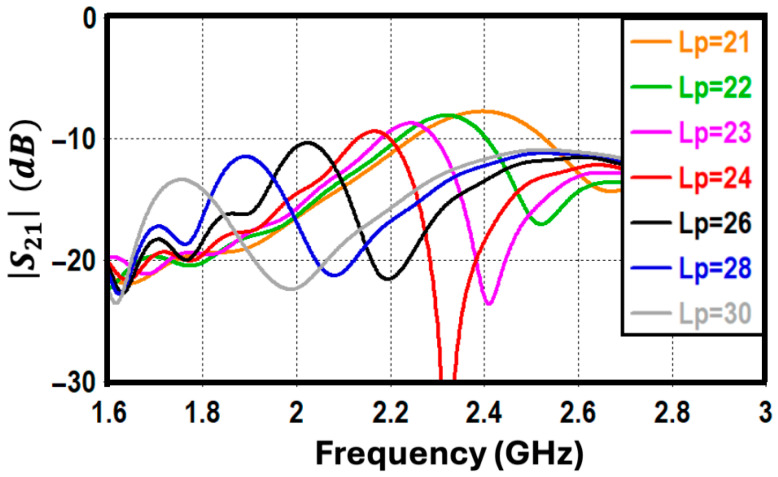
Mutual coupling coefficient (S_21_ in dB) of the proposed antenna for different parasitic element lengths (*L_p_*).

As previously noted, mutual coupling is mitigated by embedding an L-shaped parasitic element within the antenna structure. The frequency of peak isolation is directly controlled by the physical length of this component, *L_p_*. The simulated coupling responses for different *L_p_* values, plotted in Figure 3, demonstrate that the decoupling resonance shifts downward in frequency as the element is lengthened. This relationship verifies that the parasitic structure operates as a half-wavelength resonator. For instance, an *L_p_* of approximately 23 mm yields optimal isolation at 2.3 GHz, which theoretically matches the half-guided wavelength (*λ_g_*/2) at this operating frequency.

**Figure 4:**
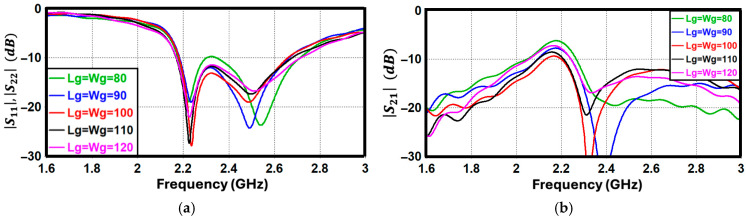
S-parameters (dB) of the proposed antenna for different ground plane dimensions (*L_g_*, W_g_). (**a**) Reflection coefficient and (**b**) mutual coupling.

To evaluate the sensitivity of the antenna to its ground plane dimensions, a parametric sweep of its overall length and width was performed. The corresponding reflection and transmission coefficients are plotted in Figure 4. As observed, the scattering parameters exhibit remarkable stability, demonstrating that the overall antenna performance is minimally affected by the ground plane area. This independence implies that the induced surface currents are concentrated predominantly on the radiating inverted-F structures rather than flowing extensively over the ground layer. Because of this inherent insensitivity, the proposed configuration is highly reliable for compact electronic devices where space limitations severely restrict the available ground footprint.

**Figure 5:**
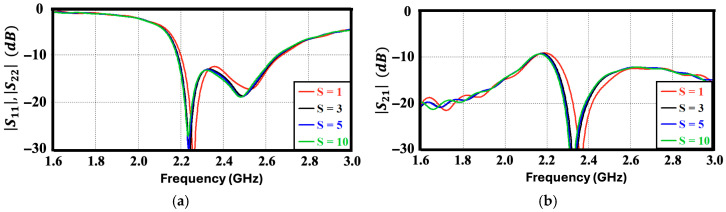
S-parameters (dB) of the proposed antenna for different substrate edges and L-parasitic spacings (*S*). (**a**) Reflection coefficient and (**b**) Mutual coupling.

To evaluate the influence of the substrate margin, the distance (S) between the L-shaped parasitic structure and the dielectric edge was parametrically analyzed. As illustrated in Figure 5, variations in this edge spacing yield no significant changes in either the reflection or coupling coefficients. This stability underscores the robust nature of the proposed antenna, demonstrating that its overall electromagnetic performance is highly resilient to variations in the available substrate clearance area.

### 2.3. Antenna Parameters for Designs with and Without a Parasitic Element

**Figure 6:**
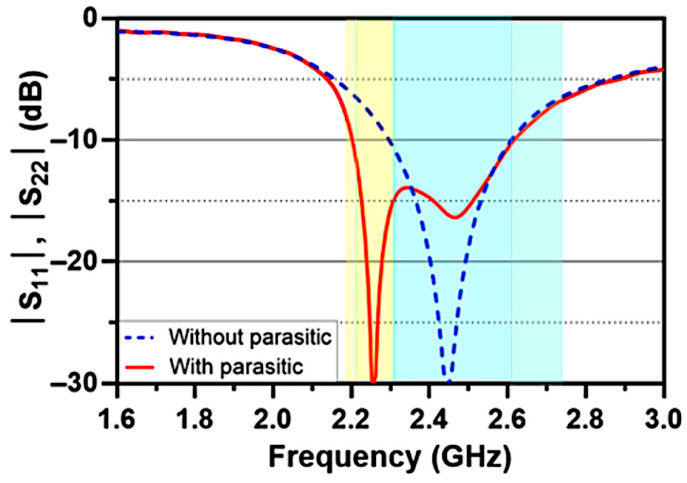
Reflection coefficient (S_11_, S_22_ in dB) of the proposed antenna with and without the L-parasitic element.

Figure 6 compares the return loss characteristics of the proposed antenna with and without the inclusion of the parasitic element. It is evident that introducing the parasitic structure enhances the impedance bandwidth by approximately 40%, primarily due to the generation of a secondary resonance around 2.25 GHz, complementing the fundamental resonance of the IFA elements near 2.4 GHz. As a result, the antenna achieves a broader operating bandwidth, covering a range from 2.2 to 2.75 GHz, with return loss values consistently below −6 dB.

**Figure 7:**
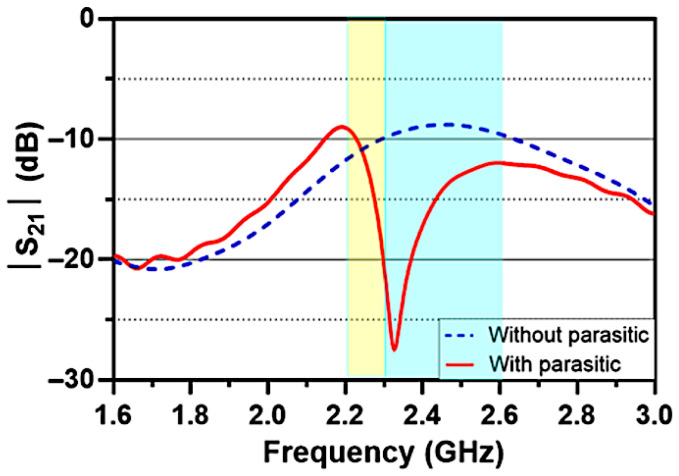
Mutual coupling (S_21_ in dB) of the proposed antenna with and without the L-parasitic element.

As illustrated in Figure 7, employing the parasitic element significantly enhances port-to-port isolation, reducing mutual coupling by nearly 15 dB compared to the reference design. This enhancement stems from the strip functioning as an efficient decoupling structure. By modifying the surface current distribution between the two ports, the parasitic element facilitates pattern steering, thereby increasing radiation pattern diversity and minimizing envelope correlation. The optimum decoupling frequency is directly dictated by the physical length of the parasitic element, which acts as a half-wavelength resonator. Adjusting this dimension, therefore, allows the isolation band to be tuned to the desired operating frequency.

**Figure 8:**
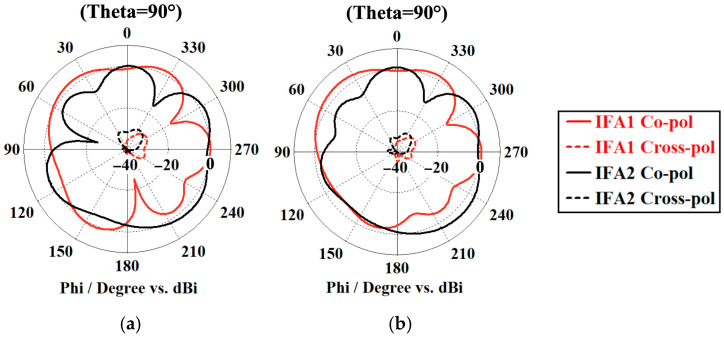
Realized gain radiation pattern (dBi) of the proposed antenna at 2.4 GHz. (**a**) The design without the parasitic element and (**b**) the antenna with the parasitic element.

A comparison of the azimuth-realized gain patterns for both IFAs, with and without the proposed parasitic element, is plotted in Figure 8. The inherent orthogonal arrangement of the radiating elements establishes foundational pattern diversity. By introducing the parasitic structure, this diversity is notably improved. The element operates as a reflecting mechanism that forces the radiation patterns to diverge. Such spatial redirection effectively drops the far-field correlation, playing a crucial role in suppressing inter-element mutual coupling.

**Figure 9:**
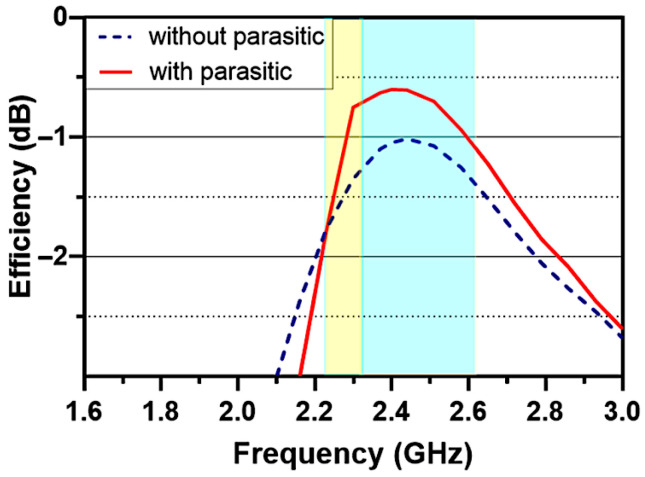
Antenna efficiency (dB) for the proposed designs with and without the L-parasitic element.

Figure 9 compares the total efficiency of the MIMO antenna with and without the presence of the parasitic element. The results indicate that incorporating the parasitic structure leads to an efficiency improvement of approximately 0.5 dB. This enhancement is primarily attributed to the suppression of mutual coupling between the two IFA elements, which reduces power losses.

**Figure 10:**
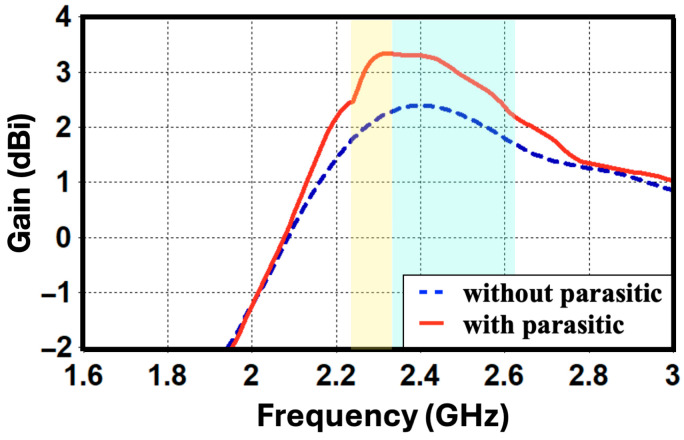
Antenna gain (dBi) for the antennas with and without the L-parasitic element.

Figure 10 presents a comparison of the peak realized gain (in dBi) versus the frequency for the antenna configurations with and without the L-parasitic element. The results indicate that incorporating the parasitic structure yields an improvement of approximately 1 dB in peak gain within the 2.4 GHz band of interest by boosting the gain from around 2.3 dBi to 3.3 dBi. This enhancement is mainly attributed to the reduction in mutual coupling between antenna elements, which consequently improves the overall radiation efficiency.

**Figure 11:**
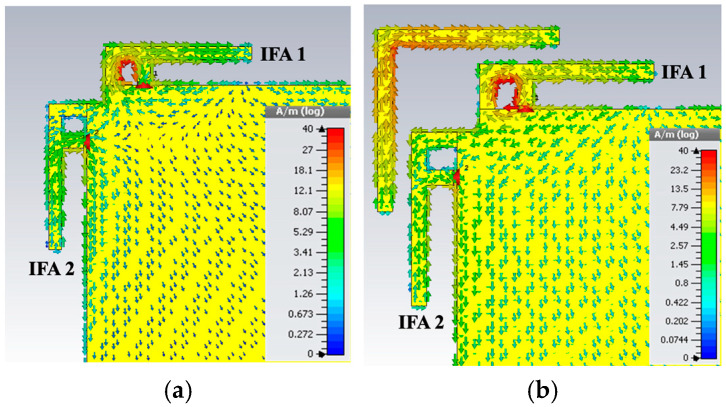
Surface current distribution of the proposed antenna at 2.4 GHz (IFA 1 is excited). (**a**) The design without the parasitic element and (**b**) the design with the parasitic element.

The surface current distributions at 2.4 GHz for both antenna configurations—with and without the parasitic element—are illustrated in Figure 11. In the absence of the parasitic element, a considerable portion of the current excited at port 1 is observed propagating toward port 2, indicating a relatively strong mutual coupling. When the L-shaped parasitic strip is introduced, it functions as a half-wavelength resonator at the frequency where coupling is minimized, with the maximum current concentrated near its center and minimal currents at its ends. This structure effectively alters the current paths on the antenna surface, disrupting the coupling mechanism and significantly reducing current transfer between the two ports, thereby enhancing isolation.

### 2.4. MIMO and Diversity Performance

To thoroughly evaluate the proposed antenna configurations—both with and without the integration of the parasitic element—several critical MIMO and diversity metrics were examined. Among these, the mean effective gain (MEG) serves as a primary indicator, quantifying the ratio of the antenna’s average received power to the total mean incident power along a specific route. An antenna’s MEG profile is fundamentally governed by the interplay between its inherent radiation characteristics and the statistical distribution of incoming waves within the operational environment. For the *i*th element in a multi-antenna array, MEG is formulated according to [7]:

(2) $${MEG}_{i}=\iint \left\{\frac{\Gamma }{1+\Gamma }G_{\theta }\left(\Omega \right)P_{\theta }\left(\Omega \right)+\frac{1}{1+\Gamma }G_{\phi }\left(\Omega \right)P_{\phi }\left(\Omega \right)\right\}d\Omega$$

where Γ is the cross-polarization power ratio, which is defined the balance between the average incident powers corresponding to the vertical and horizontal polarizations, e.g., $\Gamma =P_{V}/P_{H}$, $G_{\theta }\left(\Omega \right)$ and $G_{\phi }\left(\Omega \right)$ are the θ and ϕ components of the antenna power gain patterns, respectively, and $P_{\theta }\left(\Omega \right)$ and $P_{\phi }\left(\Omega \right)$ are the θ and ϕ components of the angular density functions of the incoming plane waves, respectively, and dΩ is the differential of the beam solid angle given by, dΩ=sinθdθdϕ.

Assuming an isotropic or uniform propagation scenario, Γ=1 and $P_{\theta }=P_{\phi }=1/4\pi$, the MEG was calculated as follows:

(3) $${MEG}_{i}=\frac{e_{tot}^{i}}{2}=\frac{e_{rad}^{i}\cdot e_{mis}^{i}}{2}$$

where $e_{tot}^{i}$ is the total efficiency of the *i*th antenna element defined by the product of its inherent efficiency, $e_{rad}^{i}$, and mismatch efficiency, and $e_{mis}^{i}$, expressed as:

(4) $$e_{mis}^{i}=1-\sum_{j=1}^{N}{\left|S_{ij}\right|}^{2}$$

The envelope correlation coefficient (ECC) acts as another vital benchmark for assessing multi-antenna efficacy, particularly within Rayleigh fading environments. This parameter characterizes the signal dependence at the receiving end under specific propagation conditions. Because pronounced channel correlation severely deteriorates MIMO throughput, maintaining a minimal ECC is essential; a lower ECC directly corresponds to reduced mutual coupling and, consequently, superior diversity operation. The ECC and the associated cross-correlation coefficient are computed using:

(5) $${ECC}_{\;ij}≅{\left|\rho_{c\;ij}\right|}^{2}$$

where

(6) $$\rho_{c\;ij}=\frac{\iint A_{ij}(\Omega )d\Omega }{\sqrt{\iint A_{ii}\left(\Omega \right)d\Omega \cdot \iint A_{jj}(\Omega )d\Omega }}$$

where

(7) $$A_{ij}=XPR{\;E}_{\theta ,i}\left(\Omega \right)E_{\theta ,j}^{*}\left(\Omega \right)P_{\theta }\left(\Omega \right)+E_{\Phi ,i}\left(\Omega \right)E_{\Phi ,j}^{*}(\Omega )P_{\phi }\left(\Omega \right)$$

where $E_{\theta }\left(\Omega \right)$ and $E_{\phi }\left(\Omega \right)$ are the θ and ϕ components of the antenna electric far-field gain patterns, respectively.

Finally, diversity gain (DG) provides an essential measure of overall MIMO robustness. This metric relies heavily on the aforementioned ECC between the antenna signals. Superior inter-element isolation inherently yields an elevated diversity gain, a relationship mathematically defined as:

(8) $$DG=10\times \sqrt{1-{\left|ECC\right|}^{2}}$$

The key MIMO and diversity performance metrics of the proposed designs at 2.4 GHz were analyzed, and the extracted results are summarized in Table 1. These parameters were evaluated assuming a statistically homogeneous propagation environment, which is commonly adopted to approximate realistic wireless channel conditions. The comparative results clearly indicate that the optimized antenna configuration employing parasitic elements provides substantial improvements over the reference design. Specifically, the optimized structure yields an enhanced diversity gain and higher multiplexing efficiency, while achieving a reduced envelope correlation coefficient. These improvements collectively confirm the superior robustness and efficiency of the proposed antenna in the multi-antenna system operation.

## 3. Fabrication and Experimental Measurements

**Figure 12:**
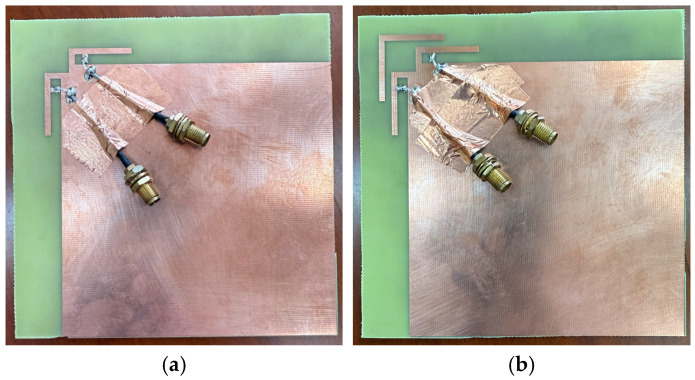
Photographs of fabricated prototypes: (**a**) the antenna without the parasitic element and (**b**) the antenna with the parasitic element.

The proposed antennas, with and without the L-shaped parasitic element, were fabricated and experimentally tested to validate the simulated performance. Photographs of the fabricated prototypes are provided in Figure 12. Both antenna configurations were realized on a 0.8 mm-thick FR4 substrate, with 50 Ω pigtails used to feed the IFA elements. To suppress unwanted cable radiation, the pigtails were grounded to the antenna’s ground plane using copper tape.

**Figure 13:**
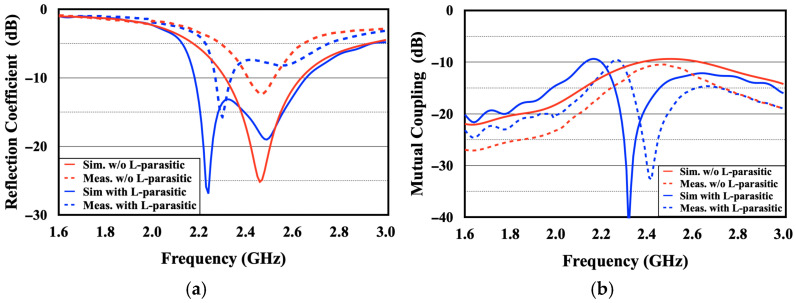
Simulated and measured S-parameters of the proposed designs. (**a**) Reflection coefficient (S_11_ and S_22_) and (**b**) mutual coupling (S_21_).

The simulated and measured S-parameters for the fabricated prototypes are depicted in Figure 13. In Figure 13a, the reflection coefficients are contrasted for both the reference and proposed antenna designs. The integration of the L-shaped parasitic structure clearly excites an extra resonance, which consequently expands the operating band. Notably, the measured 6 dB impedance bandwidth widens from a baseline of 2.33–2.60 GHz to 2.25–2.70 GHz upon adding the parasitic component, aligning with the simulation predictions. Figure 13b plots the measured mutual coupling for both setups. It is evident that the parasitic scatterer drastically mitigates coupling around 2.4 GHz, yielding an isolation enhancement of more than 20 dB. Furthermore, adjusting the physical length of this L-shaped element allows for flexible tuning of the decoupling frequency. Discrepancies between the full-wave simulations and empirical data can be primarily ascribed to manufacturing imperfections, testing environment anomalies, and the presence of the pigtail cable, which was omitted during modeling. Ultimately, the measurement campaigns successfully validate the simulated predictions, demonstrating the parasitic structure’s efficacy in boosting inter-port isolation.

**Figure 14:**
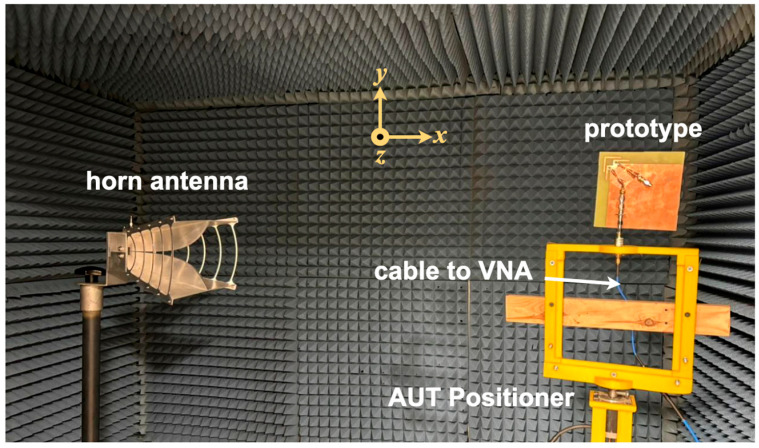
The anechoic chamber measurement setup.

To empirically validate the radiation characteristics of the fabricated prototype, experimental measurements were conducted within an RF anechoic chamber. This specialized facility successfully suppressed undesirable multipath reflections and isolated the testing environment from external electromagnetic interference, as detailed by the experimental configuration presented in Figure 14. During the characterization process, the antenna under test (AUT) was affixed to a low-permittivity dielectric positioner. Utilizing a non-conductive mount is essential to minimize structural scattering that might otherwise distort the captured radiation profiles. The AUT was subsequently aligned with a standard, linearly polarized reference antenna situated at the requisite far-field distance. Signal transmission and reception were driven by a vector network analyzer (VNA). To eliminate systematic phase and magnitude discrepancies inherent to the cables and RF connectors, a rigorous calibration sequence was applied to the entire measurement path prior to data acquisition. Ultimately, this strictly controlled methodology ensured a high-fidelity and reliable extraction of the antenna’s free-space far-field performance.

**Figure 15:**
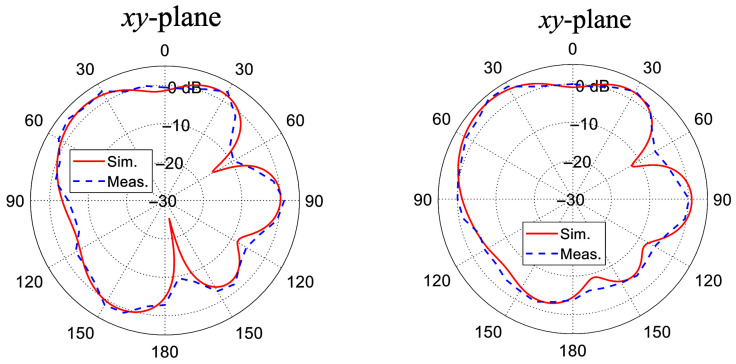

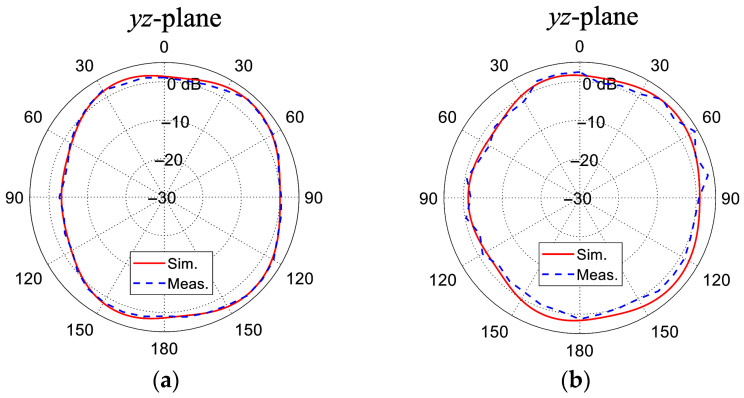
The measured radiation patterns of the fabricated antennas. (**a**) The prototype without the L-parasitic and (**b**) the prototype with the L-parasitic.

The measured far-field radiation patterns of the antenna configuration, both with and without the integration of the L-shaped decoupling structure, are illustrated in Figure 15. A comparative analysis reveals a high degree of conformity between the experimental measurements and the simulations, thereby substantiating the physical robustness and computational fidelity of the proposed design methodology. Furthermore, the measurements validate that the inclusion of the parasitic architecture yields an enhancement in the overall pattern diversity. From an electromagnetic perspective, the L-shaped component functions as a localized parasitic reflector that dynamically alters wave propagation, inducing a deliberate angular divergence between the primary radiation lobes. This engineered spatial redirection fundamentally diminishes the far-field ECC, which in turn effectively suppresses the mutual coupling.

## 4. Comparison with Related State-of-the-Art Designs

To rigorously benchmark the efficacy of the presented decoupling methodology, Table 2 delineates a comprehensive comparative analysis between the proposed dual-polarized MIMO antenna and several recently published state-of-the-art designs operating within the 2.4 GHz WLAN spectrum. The evaluation metrics encompass critical physical and operational parameters, including overall footprint, substrate material profile, mutual coupling suppression levels, decoupling mechanisms, radiation efficiency, envelope correlation coefficient (ECC), and diversity gain (DG).

As corroborated by the tabulated data, the proposed L-parasitic element technique yields an exceptional inter-port isolation exceeding 30 dB. This represents a distinct enhancement over existing methodologies—such as neutralization lines [30], RF MEMS switches [31], and electromagnetic bandgap (EBG) structures [32]—which report peak isolation levels strictly between 15 dB and 28 dB. Furthermore, unlike the complex geometries or bulky volumetric profiles observed in references [27,32], the current design leverages a highly straightforward parasitic implementation on a low-profile 0.8 mm FR4 substrate. This architectural choice ensures ease of fabrication while preserving a compact 118 × 118 mm^2^ footprint.

In terms of radiation characteristics, the proposed antenna maintains a robust total efficiency of around 87%. This markedly outperforms most of the referenced two-port planar structures (e.g., [28,30,32,34]), which frequently suffer from inherent ohmic or dielectric losses introduced by complex decoupling networks or patterned ground structures. From a spatial diversity perspective, the design exhibits an exceptionally low ECC of less than 0.0004 (derived directly from 3D radiation patterns) alongside an ideal diversity gain of 10 dB.

Consequently, this comparative assessment substantiates that the proposed dual-polarized configuration achieves a superior engineering trade-off. By delivering the highest reported isolation among the two-port planar equivalents and near-ideal MIMO diversity metrics—all while maintaining structural simplicity—the proposed antenna is established as a highly competitive and reliable solution for modern 2.4 GHz WLAN systems.

## 5. Conclusions

In this paper, a compact, dual-diversity two-element MIMO antenna system has been proposed and experimentally validated for 2.4 GHz WLAN applications. The design utilizes two orthogonally oriented inverted-F antenna (IFA) elements to inherently achieve both pattern and polarization diversity. To address the mutual coupling challenges typical of closely spaced radiating elements, a simple yet highly effective L-shaped parasitic structure was integrated into the planar layout. This element not only acts as a strong decoupling mechanism, yielding an inter-port isolation exceeding 30 dB, but also introduces an additional resonance that broadens the impedance bandwidth by 80%, covering the 2.2 to 2.7 GHz frequency range. The proposed configuration delivers exceptional MIMO performance, featuring an envelope correlation coefficient (ECC) of approximately 0.0004 and a diversity gain of nearly 10 dB. Measurements of the fabricated prototype demonstrate a strong agreement with the full-wave simulations. Consequently, with its straightforward, highly manufacturable planar geometry and robust RF characteristics, the proposed antenna stands as a highly practical solution for space-constrained, modern wireless communication systems.

## Figures and Tables

**Table 1:** MIMO parameters at 2.4 GHz for the design with and without the L-parasitic element.

MIMO Parameter	Without Parasitic	with Parasitic
Envelope Correlation Coeff. (ECC)	0.02	0.0004
Mean Effective Gain (MEG)	−3.01 dB	−3.01 dB
Diversity Gain (DG)	9.96	10.0
Multiplexing efficiency	−0.97 dB	−0.53 dB

**Table 2:** Comparison of proposed antenna performance with relative decoupling studies in the literature that operate at the 2.4 GHz WLAN band.

Reference	Dimensions (mm^3^)/Material (Height mm)	Isolation Level (dB)	Decoupling Technique	Efficiency (%)	ECC (Calc. Method)/Diversity Gain (dB)	No. of Ports/Remarks
[26]	60.2 × 60.2 × 1.6/FR4	>25 dB	Square Ring DGS	81%	<0.1 (S-param.)/9.94 dB	Four ports/compact structure and simple fabrication
[27]	160 × 160 × 14.8/RO3003	>15 dB	Dielectric Resonator	97%	<0.037 (S-param.)/9.99 dB	eight ports/complex geometry
[28]	100 × 50 × 0.8/FR4	>18 dB	Ring Resonator	29%	<0.3 (S-param.)/9.53 dB	Four ports/low thickness, large dimensions
[29]	70 × 100 × 1.6/FR4	>20 dB	Slotted Resonator	86.6%	<0.016 (Rad. Pattern)/9.99 dB	Two ports/simple geometry, dual band
[30]	135 × 80 × 0.8/FR4	>23 dB	Neutralization line	62%	<0.081 (S-param.)/9.97 dB	Two ports/complex Structure
[31]	46 × 20 × 1.6/FR4	>18 dB	RF MEMS Switches	83%	<0.2 (Rad. Pattern)/9.89 dB	Four ports/complex geometry
[32]	35 × 40 × 1.6/FR4	>28 dB	EBG Structure	67%	0.01 (S-param.)/9.9 dB	Two ports/extremely complex structure
[33]	112 × 55 × 1.6/FR4	>15 dB	Decoupling Network	75%	<0.23 (Rad. Pattern)/9.73 dB	Two ports/large dimensions, dual band
[34]	72.4 × 20 × 0.8/RO4350B	>28 dB	Patterned Ground	70.5%	<0.09 (Rad. Pattern)/9.95 dB	Two ports/medium size, complex geometry
Current study	118 × 118 × 0.8/FR4	>30 dB	L-Parasitic Element	87%	<0.0004 (Rad. Pattern)/10 dB	Two ports/compact, dual-polarized, with the highest isolation and efficiency

## Data Availability

The datasets used and/or analyzed during the current study are available from the corresponding author on reasonable request.
